# Livestock-Associated MRSA CC1 in Norway; Introduction to Pig Farms, Zoonotic Transmission, and Eradication

**DOI:** 10.3389/fmicb.2019.00139

**Published:** 2019-02-08

**Authors:** Petter Elstrøm, Carl Andreas Grøntvedt, Christina Gabrielsen, Marc Stegger, Øystein Angen, Solfrid Åmdal, Hege Enger, Anne Margrete Urdahl, Solveig Jore, Martin Steinbakk, Marianne Sunde

**Affiliations:** ^1^The Norwegian Institute of Public Health, Oslo, Norway; ^2^The Norwegian Veterinary Institute, Oslo, Norway; ^3^St. Olavs Hospital, Trondheim, Norway; ^4^Statens Serum Institut, Copenhagen, Denmark; ^5^The Norwegian Food Safety Authority, Brumunddal, Norway

**Keywords:** LA-MRSA, CC1-MRSA, One Health, pig farms, WGS, transmission, control, Norway

## Abstract

Farm animals have been identified as an emerging reservoir for transmission of livestock-associated methicillin-resistant *Staphylococcus aureus* (LA-MRSA) to humans. The low incidence of MRSA in humans and farm animals in Norway has led to the implementation of a national strategy of surveillance and control of LA-MRSA aiming to prevent livestock becoming a domestic source of MRSA to humans. In 2015, MRSA clonal complex 1 *spa*-type t177 was identified in nine Norwegian pig herds in two neighboring counties. An outbreak investigation was undertaken, and measures of control through eradication were imposed. We performed a register-based cohort study including pig herds and MRSA-positive persons in Norway between 2008 and 2016 to investigate the livestock-association of MRSA CC1, the transmission of the outbreak strain to humans before and after control measures, and the effect of control measures imposed. Data from the Norwegian Surveillance System of Communicable Diseases were merged with data collected through outbreak investigations for LA-MRSA, the National Registry and the Norwegian Register for Health Personnel. Whole-genome sequencing was performed on isolates from livestock and humans identified through contact tracing, in addition to t177 and t127 isolates diagnosed in persons in the same counties. It is likely that a farm worker introduced MRSA CC1 to a sow farm, and further transmission to eight fattening pig farms through trade of live pigs confirmed the potential for livestock association of this MRSA type. The outbreak strain formed a distinct phylogenetic cluster which in addition to the pig farms included one sheep herd and five exposed persons. None of the investigated isolates from possible cases without direct contact to the MRSA positive farms were phylogenetically related to the outbreak strain. Moreover, isolates of t177 or t127 from healthcare and community-acquired cases were not closely related to the outbreak cluster. Eradication measures imposed were effective in eliminating MRSA t177 from the positive pig holdings, and the outbreak strain was not detected in the national pig population or in persons from these counties after control measures.

## Introduction

Norway has established a unique control strategy for methicillin-resistant *Staphylococcus aureus* (MRSA) in the pig population, which includes population-wide annual surveillance in addition to contact tracing upon detection of MRSA in pig farms or farm workers. When livestock-associated MRSA (LA-MRSA) is detected in pig farms, measures of control and eradication are imposed. All findings of MRSA in humans are notifiable to the Norwegian Surveillance System for Communicable Diseases (MSIS) (Norwegian Ministry of Health Care Services, 2003), and livestock workers found MRSA-positive are offered decolonization treatment. Combined, this enables a “One Health” approach to prevent LA-MRSA from becoming established in pig farms as a domestic reservoir for zoonotic transmission to humans and the healthcare sector.

During the last decade, livestock and notably pig holdings have been identified globally as a reservoir of MRSA of importance for zoonotic transmission to humans. In Europe and North-America, LA-MRSA most often belongs to clonal complex (CC) 398 (Butaye et al., 2016). MRSA CC398 commonly causes carriage in persons occupationally exposed, and human-to-human transmission beyond household members is less frequent, a finding that is also supported by a study from Norway (Grøntvedt et al., 2016). However, recent surveillance data from the Netherlands and Denmark demonstrate an increasing frequency of LA-MRSA in humans with no reported livestock contact (Larsen et al., 2015; Bosch et al., 2016). Other lineages of MRSA have also been reported as livestock-associated, including CC9 and CC130, both commonly identified *S. aureus* clones in animals (Cuny et al., 2015b). MRSA belonging to CC1 has been recognized as a successful hospital- and community-acquired MRSA lineage in humans, but have also been reported from different livestock species, particularly *spa*-type t127 from pigs and cattle in Italy (Alba et al., 2015).

Norway has low prevalence of MRSA, but CC1 represents one of the most commonly identified clonal complexes in humans, and *spa*-type t127 has caused outbreaks in Norwegian hospitals during recent years (unpublished data from MSIS). The first detection of MRSA CC1 in Norwegian livestock occurred in 2015, with the identification of two fattening pig farms positive for *spa*-type t177 in the national MRSA surveillance program (Urdahl et al., 2016). The Norwegian Food Safety Authority (NFSA) initiated contact tracing and identified a cluster of nine positive pig farms located in the same area.

The first aim of the study was to investigate the livestock-association of MRSA CC1 t177. Secondly we aimed to investigate the extent and consequences of transmission of the outbreak strain to humans before and after control measures, and the effect of measures imposed in a One Health perspective.

## Materials and Methods

### Study Design and Population

We performed a register-based cohort study including pig holdings and persons notified with MRSA in Norway in the period between and including 2008 and 2016.

### Surveillance and Control of MRSA in Pigs

Norway has since 2011 conducted active MRSA surveillance of the pig population, which since 2014 includes the majority of pig herds (Urdahl et al., 2014, 2016, 2017). In addition to the national surveillance program, contact tracing from MRSA-positive pig herds or MRSA-positive persons with direct contact with pig herds was performed. Epidemiological data were collected through questionnaire by personnel from NFSA from herds identified, as described by Grøntvedt et al. (2016).

In the Norwegian strategy MRSA was defined as livestock-associated if it was belonging to a *spa*-type previously described as LA-MRSA, or if investigations demonstrated persistence and transmissibility in at least one pig farms. If MRSA was detected only in a low proportion of the samples analyzed, or in a single pig farm, and the *spa*-type was not previously described as LA-MRSA, the farm was subject to longitudinal sampling until sufficient evidence on persistence and transmissibility was supported or not. Thus, the definition of LA-MRSA in Norway is dependent on the epidemiology of identified *spa*-types and not restricted to CC398 strains.

During 2015, the surveillance program included specialized finisher pig herds (with an annual production of >70 slaughtered pigs) in addition to genetic nucleus and multiplier pig herds (821 pig herds in total) (Urdahl et al., 2016). Sampling in surveillance and contact tracing was conducted by personnel from the NFSA and included pooled swab cloth samples from pigs and farm environment, as previously described (Grøntvedt et al., 2016).

The NFSA imposed restrictions upon detection of MRSA reported by the Norwegian Veterinary Institute (NVI). The initial restrictions banned trade of live pigs, other than directly to slaughter. Upon confirmation of LA-MRSA, the NSFA imposed depopulation through slaughter or culling of MRSA positive pig herds. Following depopulation, the farm owner was responsible for thorough washing and disinfection of farm premises. After a mandatory down-time, the farm buildings were inspected and sampled by the NFSA. If MRSA was not detected in these samples, the farms were repopulated with pigs from MRSA-negative herds. Follow-up samples after repopulation were collected ~2 weeks before slaughter of the first batch of fatteners from the fattening pig farms, and for the sow farm after 3, 6 and 9 months. In case of MRSA detection in follow-up samples, this would reinitiate the imposing of restrictions and measures to eradicate MRSA.

### MRSA Investigations in Humans

All persons diagnosed with MRSA are reported to MSIS at the Norwegian Institute of Public Health (NIPH). Information registered in MSIS includes demographical, epidemiological, and clinical data reported by the treating clinician, together with information on the bacterial isolates reported by the primary laboratories and the Norwegian MRSA reference laboratory (Blomfeldt et al., 2017).

In this study, we included all persons reported with MRSA t177 or t127 to MSIS and living in the two counties where the outbreak of MRSA t177 in pig farms took place. Using the national unique personal identification code for all persons in Norway, we merged data from MSIS with data collected in the outbreak registry for LA-MRSA at NIPH, the National Registry and the Norwegian Register for Health Personnel.

Data from MSIS included name, age, sex, municipality and county of residency, country of birth for both the persons notified and their parents, time in Norway, occupation, MRSA sample material, MRSA CC and *spa*-type, indication of sampling, clinical status at the time of notification, type of health care facility where MRSA was diagnosed, possible place of infection and unique outbreak code identifying persons that were linked to reported MRSA outbreaks. Data from the outbreak registry for LA-MRSA included name, identification of the farm where the person was working or residing, indication of MRSA sampling (occupationally exposed or household member of a person diagnosed as LA-MRSA positive), date and the result of MRSA sampling (positive or negative). Data from the National Registry included name, address of residency, name and address of parents and/or children, and if applicable the date of death and dates of immigration to or emigration from Norway. The Register for Health Personnel included name, type of health- or veterinary education, type and date of work license or authorization.

MRSA t177 was identified in pig farms located in five municipalities within two adjacent counties. In order to identify transmission of MRSA from humans to pig herds and from farms to the public, we investigated all persons diagnosed with MRSA t177 and residing in the two counties. Due to close genetic relationship between t177 and t127 (www.spaserver.ridom.de) and the previous reports of t127 in livestock (Battisti et al., 2010; Alba et al., 2015), we also included a sample of relevant cases of MRSA t127 to investigate both the genetic relatedness of isolates and whether notified persons with t127 could be connected to the outbreak. Thus, we defined possible human cases in the outbreak as persons notified with MRSA t177 and with a home address within the two counties; or persons notified with MRSA t127 and with a home address within the five municipalities and not reported infected abroad; or persons notified with MRSA t127 and registered as veterinarian or pig worker and with a home address within the two counties; or persons notified with MRSA t177 or t127 and registered as family members (children or parents) of a possible case (persons meeting the other criteria), independent of place of residency.

We defined confirmed human cases of the farm outbreak to be persons defined as a possible case and with a MRSA isolate belonging to the outbreak cluster based on the results of the whole-genome sequencing (WGS).

### Bacteriological Analysis

All samples from pigs and pig holdings were analyzed for MRSA by NVI as described in the protocol by EFSA (Efsa, 2012). Human and animal MRSA isolates were confirmed and genotyped at the Norwegian MRSA reference laboratory, using a multiplex PCR targeting the *mecA, spa*, and Panton-Valentine leucocidin genes (Stegger et al., 2012), followed by Sanger sequencing of the *spa* amplicon (www.spaserver.ridom.de). Antibiotic susceptibility profiles were obtained using disk diffusion and minimum inhibitory concentration (MIC) assays according to EUCAST guidelines (www.eucast.org).

The following isolates were subject to whole-genome sequencing (WGS): isolates from animals or environment in each farm (coded as S and F in **Figure 4**); isolates from persons defined as possible cases (coded as H in **Figure 4**); isolates from a convenience sample of t127 isolates identified in the two counties, distributed by community-associated cases (coded as C in **Figure 4**) and health care associated cases (coded as HC in **Figure 4**).

In total 38 isolates were sequenced, distributed by nine isolates from pig herds and one isolate from sheep included in the outbreak investigation, and 28 isolates from 27 persons. Two MRSA isolates from one person were included in the sequencing because the person was found positive with the outbreak strain before and during the outbreak investigation.

MRSA isolates sequenced were treated with proteinase K (2 mg/mL) and lysostaphin (0.1 mg/mL) for 15 min with shaking at 37°C, before heating for 15 min at 65°C. Genomic DNA was isolated using the EZ1 DNA tissue kit on an EZ1 Advanced XL instrument (Qiagen). Sequencing libraries were prepared using the Nextera XT sample prep kit, and were sequenced on the MiSeq platform with 300 bp paired-end reads (MiSeq Reagent Kit v3) (Illumina). Raw data was quality controlled using FASTQC 0.11.5 (Babraham Bioinformatics) and filtered/trimmed using Trimmomatic 0.32 (Bolger et al., 2014), before *de novo* assembly using the SPAdes assembler 3.5.0 (Bankevich et al., 2012). Draft genomes were annotated using Prokka 1.12 (Seemann, 2014). The core and accessory genome was defined and a core genome alignment produced using Roary 3.6.8 (Page et al., 2015). The core genome alignment was used as reference for extracting core genome SNPs using SNP-sites 2.1.3 (Page et al., 2016). Substitution models were evaluated using Smart Model Selection 1.8.1 and a maximum-likelihood phylogeny constructed using PhyML 3.1 using the GTR substitution model (Criscuolo, 2011). ABRicate software was used for *in silico* prediction of resistance- and virulence genes present in the isolates (https://github.com/tseemann/abricate). Threshold for identification and coverage was 90 and 60%, respectively. Prediction of SCC*mec*-type and prophage sequences was performed using SCC*mec*Finder 1.2 (Kaya et al., 2018) and PHASTER (PHAge Search Tool Enhanced Release) accordingly (Arndt et al., 2017). The datasets generated for this study are available from the NCBI Sequence Read Archive accession number SRP159059.

### Approvals

The study received ethical clearance from the Regional Committee for Medical and Health Research Ethics (2017/2528/REK sør-øst A), and approvals for the use and merging of register data from the Norwegian Institute of Public Health (MSIS), the Norwegian Tax Administration (National Registry) and the Norwegian Directorate of Health (Register for Health Personnel).

## Results

### Description of the Outbreak in Pig Herds

**Figure 1:**
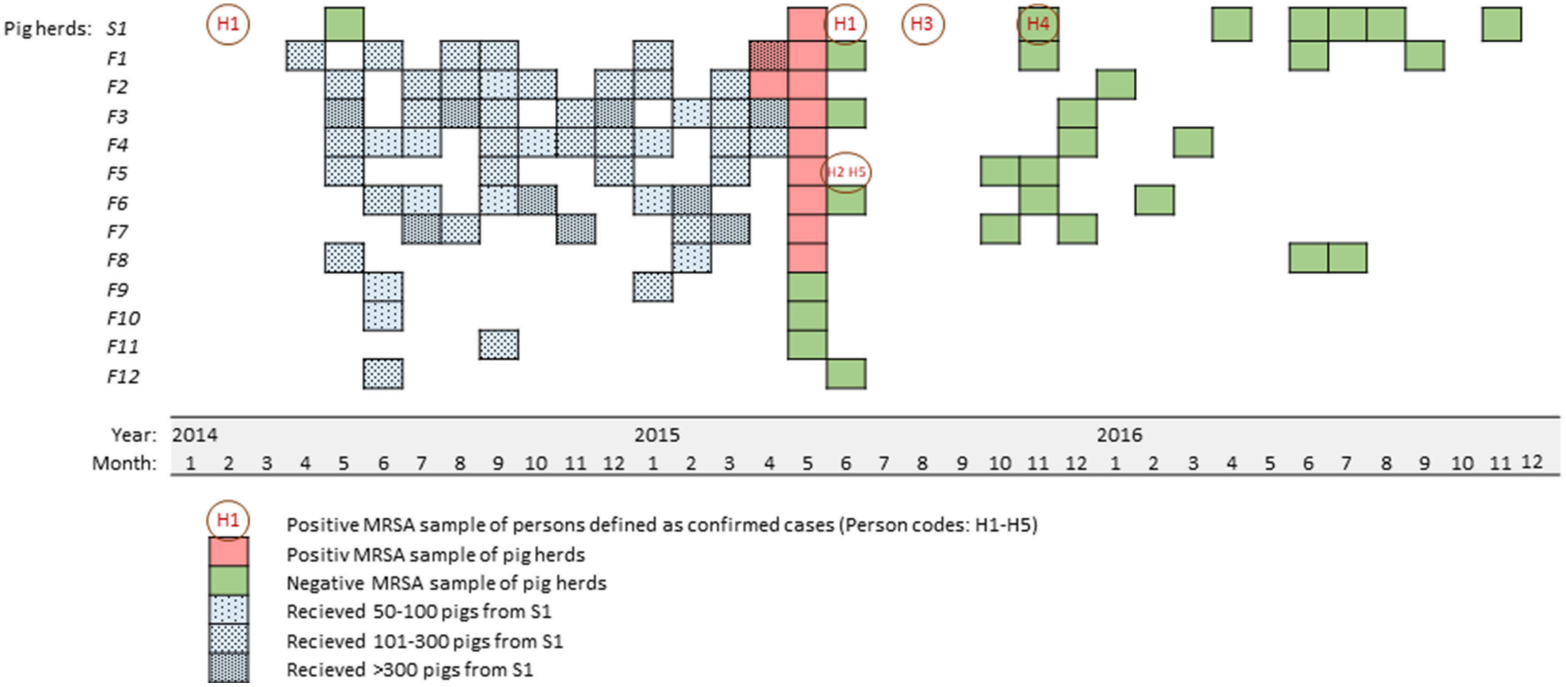
Time line showing the number and time of pig transfers from the sow farm (S1) to the fattening pig farms (F1-F12, blue squares) during 12 months before MRSA were identified in nine farms (red squares). The figure also show the time and results of MRSA sampling of humans with an epidemiological link to the farms (orange circles), and follow-up sampling of pigs and environments (green squares).

In April 2015, the initial findings of MRSA CC1 t177 in Norwegian pig holdings were made in samples from two specialized fattening pig farms (Urdahl et al., 2016). Contact tracing revealed that one grower pig producing sow farm had supplied both these fattening pig farms. Follow up sampling detected MRSA in samples from the sow farm. The sow farm did not introduce pigs from other farms and had tested negative in the MRSA surveillance program in 2014 (Figure 1). In addition to delivering pigs to the two specialized fattening pig farms, it had supplied grower pigs to 10 other fattening pig farms during the last 12 months. Among these, samples from six farms were found MRSA positive, resulting in nine MRSA positive pig farms in total. MRSA CC1 t177 was the only MRSA *spa*-type detected in samples from these farms.

All the MRSA positive fattening pig farms had received grower pigs from the sow farm on several occasions (range 2–10 monthly deliveries per year, 66–322 pigs per farm per month) during the last 12 months. In contrast, the MRSA negative pig farms had received either a single delivery of 100–151 pigs per delivery (three farms) or two deliveries of 100–199 pigs per delivery (one farm). The latest delivery of pigs to a farm found negative for MRSA occurred 3 months prior to the initial detection of MRSA in the two fattening pig farms, while all the farms found positive for MRSA had received pigs from the sow farm during the last 3 months before the initial detection (Figure 1).

Of the nine MRSA positive pig farms, five farms also kept other animals, including sheep, cattle, chicken, dogs, and cats. MRSA was only detected in samples from a single sheep herd housed in the same farm building as positive pigs. The sheep were on a separate floor directly above the pig compartments, but the ventilation system connected the two floors.

### Effect of Eradication in Farms

The time from confirmation of MRSA in the pig farms, to the first follow up sampling of environment after decontamination/eradication, was 11 months for the sow farm and ranged from four to a maximum of 13 months for the fattening pig farms (mean: 6 months). All the farms were followed up as described, and MRSA was neither detected in follow up environmental samples after decontamination/eradication, nor in any samples of animals or environment after repopulation (Figure 1). Thus, we concluded the MRSA eradication to be successful in the first attempt in both the sow farm and in all eight fattening pig farms. The sheep were followed up during and after the grazing season and those that were persistently MRSA positive, were slaughtered.

### Epidemiology of MRSA t177 and t127, in Humans

In total, 555 persons were diagnosed with t177 or t127 in Norway in the study period. Among these, 97 had a home address in the two counties and 15 met the epidemiological definition of possible cases in the outbreak. The persons not meeting the definition of possible cases were all living in other municipalities than the MRSA positive farms and had no known epidemiological link to the farms (not working with animals nor in family with persons living or working on the farms). During the outbreak investigation, 65 persons were included in the case tracing, of whom five where diagnosed with MRSA, three with CC1 t177, one with CC5 t002 and one with CC398 t2974. Two and 5 months after the case tracing two household members of a worker in one the MRSA positive farms were diagnosed with MRSA CC1 t177, giving a total of five persons diagnosed with t177 and an epidemiological linkage to the farms.

**Figure 2:**
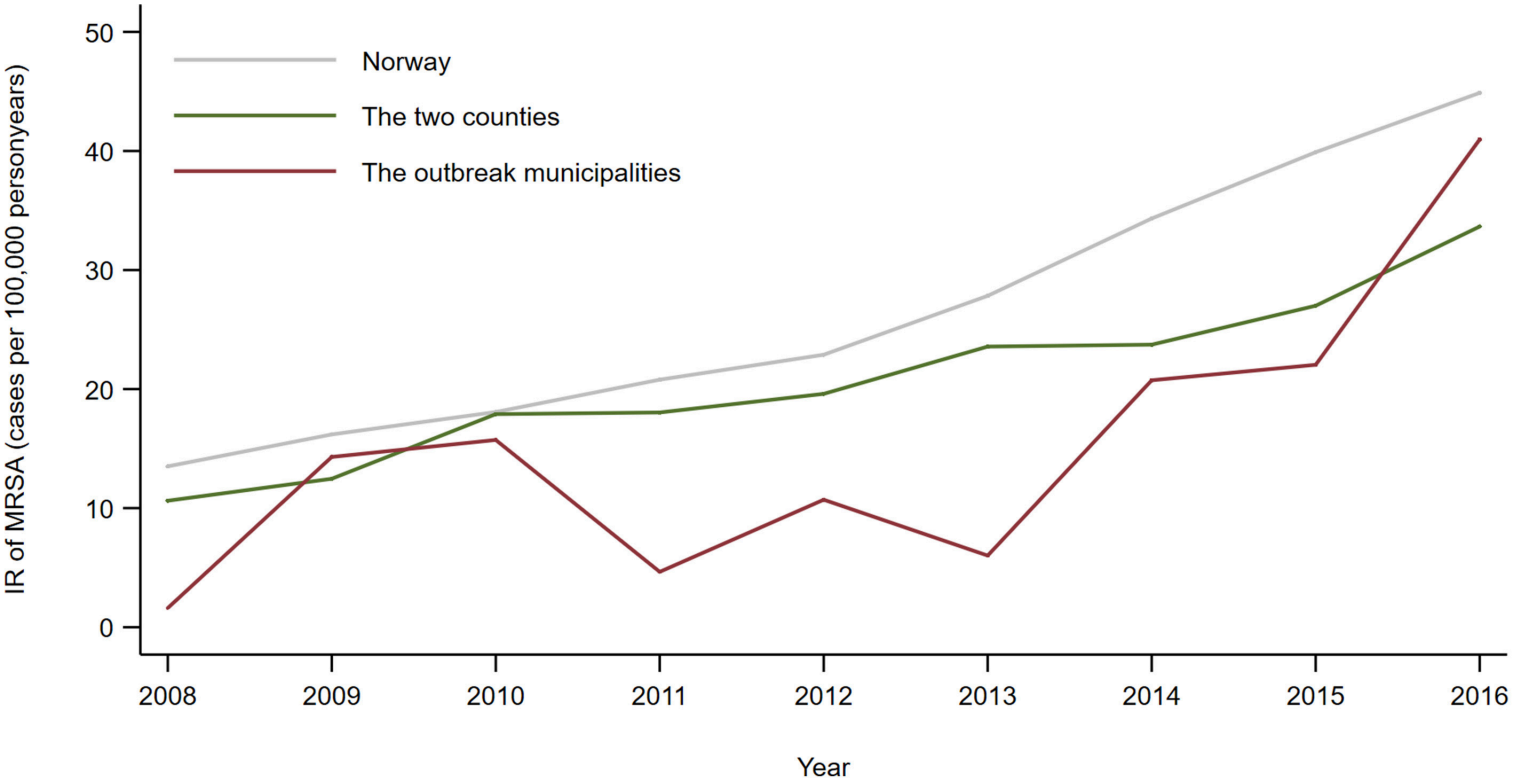
Annual incidence rate (number of notified cases per 100,000 person years) of all persons notified with MRSA 2008–2016, by country, counties and municipalities affected by the outbreak of MRSA t177 in pig farms.

Both the two counties and the five municipalities where the outbreak of MRSA t177 in farms took place had a lower annual incidence rate (IR) of persons notified with MRSA than the mean IR for Norway (Figure 2). From 2013 to 2017 the difference in IR between the counties and the whole country were statistical significant, with an incidence rate ratio (IRR) of 0.85 (95% CI: 0.73–0.98) in 2013 and 0.75 (95% CI: 0.67–0.84) in 2017.

During the study period, only 13 persons were notified with MRSA t177 in Norway, including seven in the two counties of whom six were registered residents in the municipalities with MRSA t177 in pig farms.

**Figure 3:**
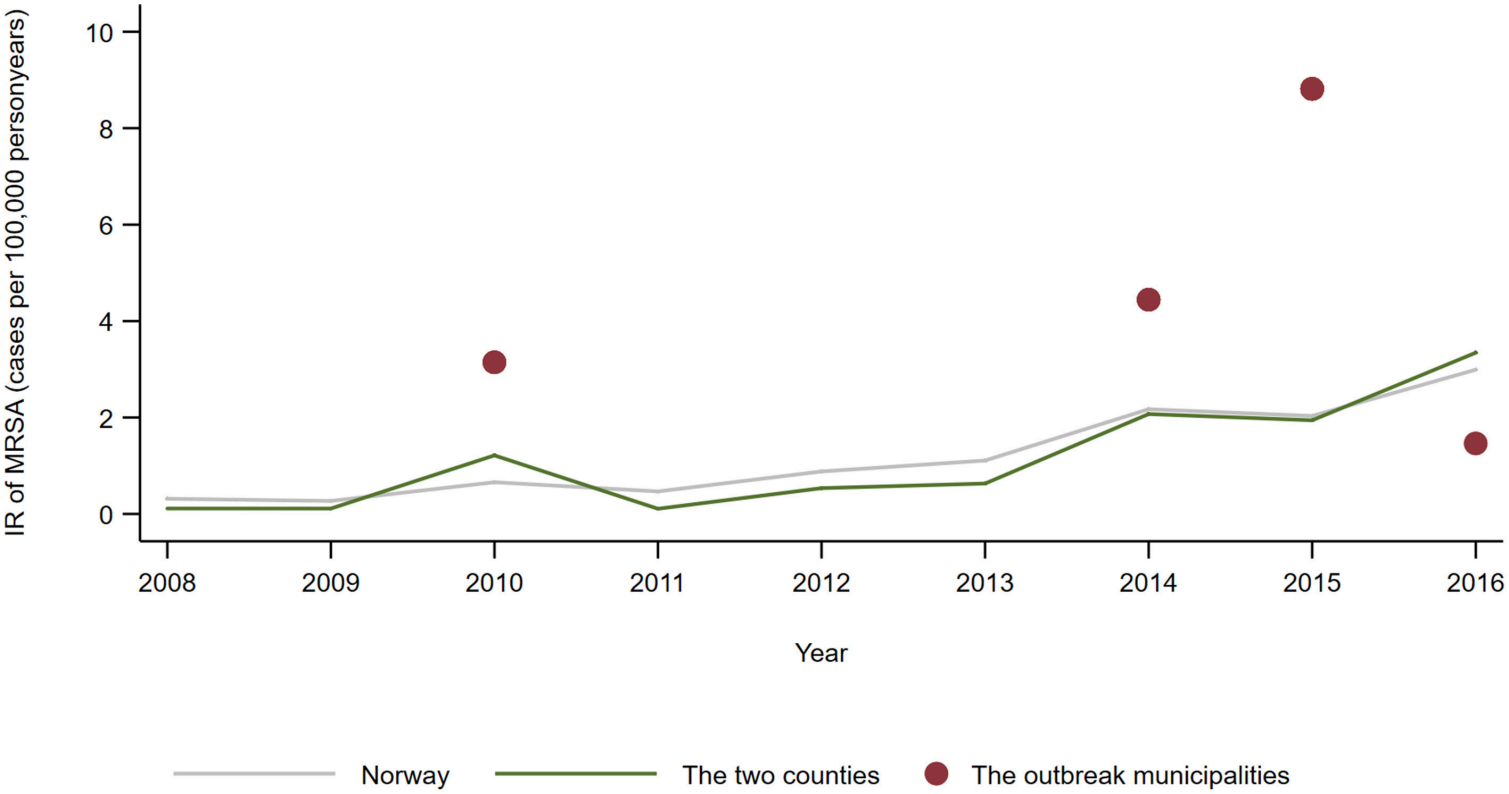
Annual incidence rate (number of notified cases per 100,000 person years) of persons notified with MRSA t177 or t127 in 2008–2016, by country, counties and municipalities affected by the outbreak of MRSA t177 in pig farms.

MRSA t127 was the fifth most often identified *spa*-type in persons in Norway in the study period, and the fourth most common in the two counties. In total, 37 different *spa*-types were notified for persons living in the five municipalities with MRSA positive farms, and t127 (*n* = 7) and t177 (*n* = 5) were among the four most common *spa*-types. The shared annual IR of t127 and t177 were higher in the outbreak municipalities than for the whole country in 2010, 2014, and 2015, and with a significant difference in 2015 (IRR 4.34; 95% CI: 1.56–9.76) (Figure 3).

The sex and age distribution of persons notified with MRSA in the counties were in line with all persons notified in Norway, both for MRSA in general and for those diagnosed with t177 or t127. Around half of all persons diagnosed with MRSA t177 or t127 were Norwegian citizens. Based on the information notified by the clinicians we assessed that persons infected in Norway constituted 38, 43, and 50% of the persons diagnosed with these *spa*-types, living in Norway, in the counties, or in the municipalities, respectively.

### Disease Burden

When comparing all notified cases of MRSA in the study period, we found a significant lower odds of being reported with an MRSA infection for persons notified with the *spa*-types t177 or t127 than for those notified with other *spa*-types (Table 1). However, persons notified with t177 or t127 had higher odds of being reported as inpatients in hospitals, including intensive care units. We found no significant difference in persons with t177/t127 compared to those with other *spa*-types, in the risk of being notified with bacteremia or reported dead within 30 days after MRSA-bacteremia (Table 1).

**Table 1:** Reported morbidity, mortality and hospitalization in persons notified with MRSA t177 or t127 in Norway compared to persons notified with other MRSA spa-types.

**Burden in persons notified with MRSA**	**Cases (%)**	**Controls**	**OR (95% CI)**
Infections in patients with non t177/t127	5,181 (45)	6,284	1
Infections in patients with t177/t127	200 (36)	355	0.68 (0.57–0.82)
Inpatients with non t177/t127	2,431 (21)	9,034	1
Inpatients with t177/t127	151 (27)	404	1.39 (1.14–1.69)
Inpatients in intensive care units with non t177/t127	74 (0.7)	11,391	1
Inpatients in intensive care units with t177/t127	17 (3.1)	538	4.86 (2.67–8.39)
Bacteremia in patients with non t177/t127	108 (0.1)	11,357	1
Bacteremia in t177/t127	8 (0.1)	547	1.54 (0.64–3.16)
30 days all-cause mortality after bacteremia in patients with non t177/t127	23 (18)	108	1
30 days all-cause mortality after bacteremia in patients with t177/t127	1 (11)	8	0.59 (0.01–4.77)

*Relative risk is calculated as odds ratio (OR) and with 95% confidence intervals (CI)*.

### Genetic Analysis of Isolates

**Figure 4:**
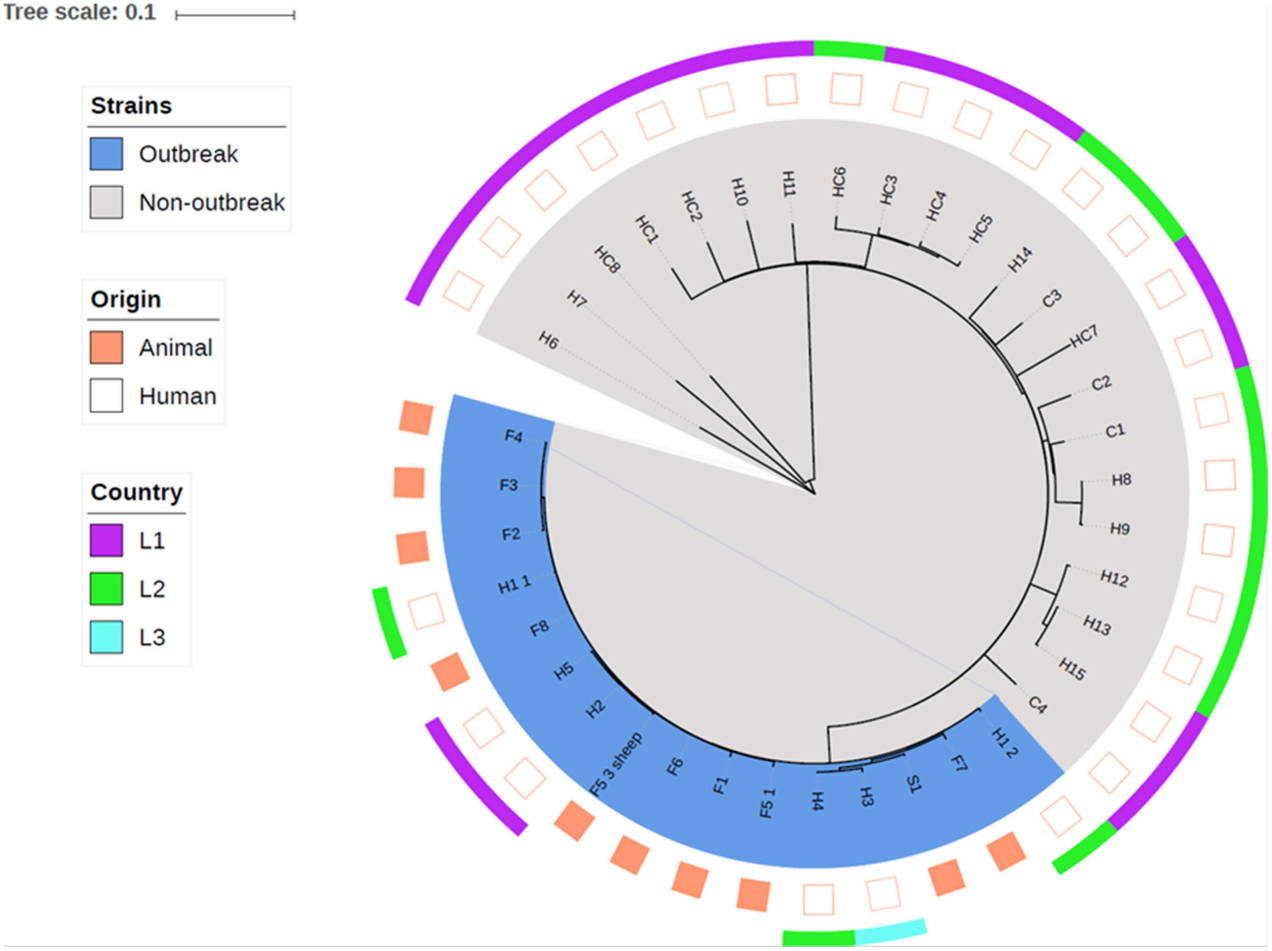
Phylogeny based on core genome alignment of human and animal MRSA CC1 t177 and t127 isolates in two adjacent counties in Norway. Animal and human isolates clustering in the investigated farm outbreak are colored in blue. The country codes indicate were the persons are born, which include Norway and two other European countries.

Whole-genome sequencing and core genome phylogeny revealed that all MRSA-positive pig herds (S1, F1–F8) as well as all MRSA t177 positive persons directly linked to these farms (H1–H5) formed a distinct phylogenetic cluster (Figure 4). Within the outbreak cluster, strains displayed from 1 to 22 core genome SNPs. Of the possible cases with no direct link to the farms (H6–H15), none were found to be closely related to the outbreak strains. Similarly, the cases of MRSA t127 from healthcare (HC1–HC8) and the community (C1–C4) did not appear to be closely related to the outbreak cluster, with ≥74 core genome SNPs distinguishing between the outbreak cluster and the nearest neighbor. One isolate of MRSA t177 with no epidemiological link to the outbreak was clearly dissimilar from the t177 strains in the outbreak cluster (≥441 SNPs).

Among the MRSA t127 isolates, we identified three small clusters in the two counties. These three clusters included patients and health care workers in an outbreak reported from a nursing home (HC3–HC6), persons with an address in the same municipality as one of the farms (H8, H9), and two persons registered as veterinarians and one of their family members (H12, H13, H15). None of the isolates in these clusters were closely related to isolates in the farm outbreak.

The core genome phylogeny (Figure 4) showed that the human MRSA t177 strains clustered closely together with the animal MRSA t177 strains, indicating that there was no clear distinction between human and animal strains. Examination of the accessory genome furthermore revealed little variation in genetic content and no specific genes which could distinguish between either the human and animal MRSA t177 strains or between the outbreak and non-outbreak strains.

**Figure 5:**
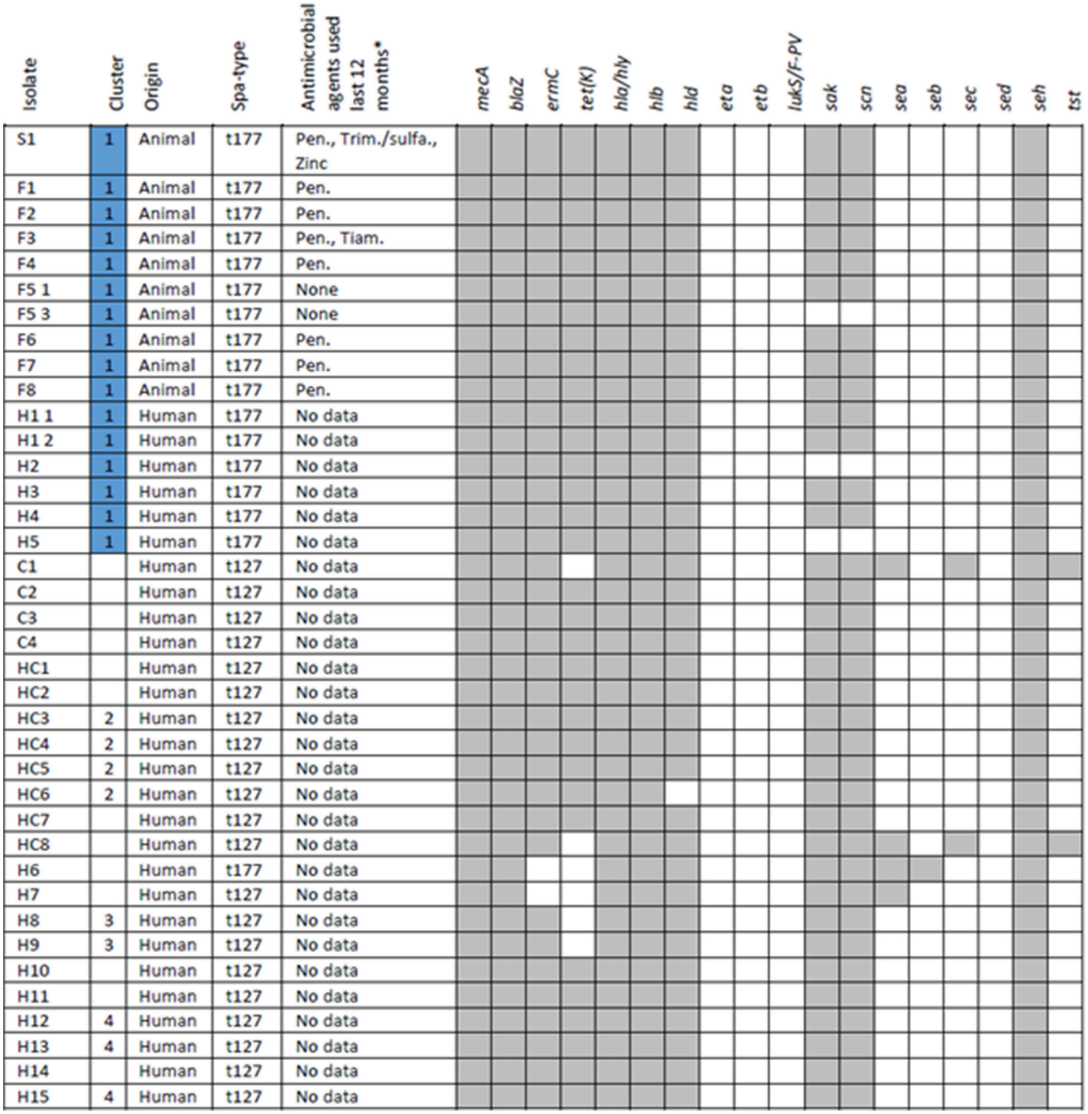
Animal and human isolates with information on spa-type, exposure to antimicrobial agents and selected antimicrobial resistance and virulence genes. ^*^Antimicrobial agents: Pen., Procaine benzylpenicillin; Trim., Trimethoprim/sulfadiazine; Zinc, Zinc oxide; Tiam., Tiamulin.

Susceptibility testing of the MRSA t177 outbreak strains showed resistance against erythromycin and tetracycline in addition to beta-lactams, including cefoxitin, which was in accordance with *in silico* resistance prediction (Figure 5). SCC*mec* prediction indicated that the outbreak strains had SCC*mec* type IVa (2B). Prediction of prophage sequences showed presence of a prophage similar to phiN315, which encoded genes (*sak, scn*) related to host immune modulation and human colonization (Van Wamel et al., 2006). Regarding virulence factors other than *sak* and *scn*, presence or absence of selected *S. aureus* toxin genes are shown in Figure 5.

Data on reported usage of antimicrobial agents were available from all nine MRSA positive pig herds (Figure 5). The piglet producing sow herd and seven fattening pig herds reported having used procaine benzylpenicillin injectable during the last 12 months. In addition, the piglet producing sow herds also reported having used trimethoprim/sulfadiazine injectable and zinc oxide per orally, and one of the fattening pig producers reported having used tiamulin (unknown route of administration). The remaining fattening pig producer reported not having used any antimicrobial agents during the last 12 months. Data on the usage of antimicrobial agents among persons epidemiologically linked to the outbreak was not collected.

## Discussion

This study confirms that some MRSA types within CC 1 have the potential for establishment and spread in livestock. Identification of MRSA CC1 among pigs has been described in other countries (Efsa, 2009; Battisti et al., 2010), but there are few other European studies that explore and describe the potential of livestock transmission of MRSA clonal complexes other than CC398.

We found that the t177 strains epidemiologically linked to the outbreak were closely related and with little variation in the genetic content. None of the other sequenced isolates (t177 or t127) clustered with the outbreak isolates.

Several studies of LA-MRSA have found differences in the gene content in human and animal isolates, indicating how MRSA strains have adapted to different hosts (Hallin et al., 2011; Price et al., 2012). Results from these studies have been used to form and test hypotheses that LA-MRSA CC398 strains are different from human MRSA strains, with a higher capacity of establishing and transmitting within animal herds and at the same time less potential for spread and capacity of causing infections in the human population (Hetem et al., 2013; Van De Sande-Bruinsma et al., 2015). In this study, we have showed that one of the MRSA strains commonly found in humans also has the potential of transmission to and between livestock. However, we did not find any specific genes or differences in the gene content between animal and human isolates that can explain the livestock association. The isolates contained a prophage similar to phiN315, which encoded genes (*sak, scn*) related to host immune modulation and human colonization. Presence of this prophage has been linked to *S. aureus* isolates adapted to human hosts. It is uncommonly found among LA-MRSA isolates from animals probably due to loss of the phage in the livestock reservoir (Cuny et al., 2015a). In our material, the patients diagnosed with t177 or t127 had an overall lower risk of infection compared with patients identified with other MRSA strains. However, the risk of MRSA bacteremia was not significantly different, and the risk of hospitalization and being inpatient in intensive care units were increased for patients positive of MRSA t177 or t127.

The mechanisms of host adaptation of *S. aureus* including LA-MRSA has been studied in several host species (Ballhausen et al., 2017; Richardson et al., 2018). In the present study however, the time from introduction to detection of MRSA and subsequent eradication was only a few months (Figure 1). This short time may have influenced the degree of host adaptation of the isolates. Nonetheless, the study demonstrates that this MRSA strain without known specific livestock adaptations may have the potential to establish and spread in livestock. The findings in this study highlights the need for a broad definition of LA-MRSA and an approach on prevention and control of LA-MRSA that include clonal complexes other than CC398. When MRSA in livestock not only can be defined as certain types of CC398, but also include MRSA strains that already are common in the human population, it becomes more difficult to prevent introduction from humans to pig herds. The consequences of zoonotic transmission to people working with animals and the threat LA-MRSA pose on public health may also be larger than previously assessed in studies only focusing on MRSA CC398 (Hetem et al., 2013; Van De Sande-Bruinsma et al., 2015).

Similar to what we previously found in a Norwegian study of LA-MRSA CC398 (Grøntvedt et al., 2016), we managed in this study to find a strong indication on how MRSA was introduced into the pig herd. A worker tested positive with MRSA t177 before starting working in the farm. Two months later, the herd was still MRSA negative when tested in the ongoing national surveillance of LA-MRSA in pigs. However, 1 year later both animals and pig workers in the sow herd and in farms receiving pigs from the sow herd, were diagnosed positive with the same MRSA strain.

The isolates linked to the outbreak in livestock showed resistance to tetracycline and erythromycin. Other studies have reported both resistance to tetracycline and erythromycin in LA-MRSA isolates and widespread use of tetracycline in production animals (Monaco et al., 2013; Larsen et al., 2016). All use of antimicrobial agents to food producing animals in Norway must be prescribed by veterinarians. Mainly narrow spectrum antimicrobial agents were reported used in the farms included in this study, and benzylpenicillin was the most commonly used antimicrobial. No herds reported having used tetracyclines or macrolides during the last 12 months. It is thus unlikely that usage of these substances contributed to the establishment and spread of MRSA CC1 in these herds.

For whole-genome sequencing, we included isolates from all persons diagnosed with MRSA t177 in the two counties, together with all pig workers in the counties and all persons living in the five outbreak municipalities notified with MRSA t127. Despite this comprehensive comparison of MRSA isolates in a period of 6 years before the outbreak started and one and a half year after control measures were imposed, we did not find any isolates that were related to the outbreak beside isolates from persons working with the MRSA-positive animals and their household members. This result, together with the fact that all samples from the farms after eradication measures were negative, show that further spread was effectively controlled. Although the pig herds in Norway are tested regularly for MRSA in the surveillance program, MRSA CC1 has not been found in other pig herds in the country neither before nor after this outbreak.

The four fattening pig farms found MRSA negative during contact tracing had all received fewer grower pigs than the positive fattening pig farms. In addition, none of them had received any pigs during the last 3 months before the time of detection in the sow farm. This suggest that the four negative pig farms had not received MRSA positive pigs either because of the total number of pigs delivered or the time of transfers. Alternatively, if these pig farms had received MRSA positive pigs, herd management routines may have contributed to MRSA eradication, or MRSA did not become established in these four farms. These findings are similar to those in a previous study of LA-MRSA CC398 in Norway (Grøntvedt et al., 2016).

Norway is a country with a very low incidence of MRSA in both the human and animal population. All MRSA diagnosed in humans are notifiable and all pig herds are regularly tested for MRSA. Additional surveys are also being done in other animal populations (https://www.vetinst.no/en/surveillance-programmes/norm-norm-vet-report). This constitutes the major strengths of both the efficacy of the Norwegian LA-MRSA strategy and regarding completeness of data in this study. The comprehensive surveillance and the low MRSA incidence makes it possible to collect and analyze epidemiological information of all human and animal MRSA cases, and to compare isolates through whole-genome sequencing.

Persons can be asymptomatic carriers over several months without being tested and identified MRSA positive (Larsson et al., 2011). Thus, the outbreak strain may have been spread to more persons than those notified to MSIS. However, persons with an MRSA infection will normally be tested and diagnosed, and pig herds have been tested on regular basis since 2013. This means that Norwegian authorities have a unique overview and control of the MRSA status in both humans and animals. The findings of MRSA in pig herds of both CC398 and CC1 have been few and all identified outbreaks have been effectively controlled (Urdahl et al., 2014, 2016, 2017, 2018; Grøntvedt et al., 2016).

Because the MRSA incidence in Norwegian livestock is very low, farm workers in Norway are not screened for MRSA on admission to hospitals based on occupation. In other countries with a low incidence of MRSA in humans, where livestock have become an increasing domestic reservoir of MRSA to the public, such prevention and control measures in hospitals are implemented as a permanent routine in order prevent spread of MRSA to vulnerable inpatients (Van De Sande-Bruinsma et al., 2015; The Danish Health Authority, 2016). As long as only a few pig herds are found MRSA positive, the resources used to eradicate MRSA in the farms are less work- and cost-demanding than routinely MRSA screening and preliminary single room isolation of all pig workers or their household members on admission to hospitals (Norwegian Food Safety Authority, 2016).

In April 2018, the Norwegian government implemented regulations that makes it compulsory for all persons potentially exposed to MRSA to use personal protective equipment to prevent transmission of MRSA from humans to pigs, unless they are tested and found MRSA negative. Although this has also previously been a recommendation, this requirement will probably increase the likelihood of finding and treat MRSA carriers before they start working with pigs. Actions supportive of the national strategy against LA-MRSA are imposed whenever MRSA of any genotype are identified in pigs or in persons in contact with the pigs.

## Conclusions

In this study, we identified CC1 t177 as an MRSA strain with a clear potential of establishment and spread in livestock. We found no significant differences in the gene content between isolates from animals and humans or between the outbreak and non-outbreak MRSA isolates. This demonstrates that LA-MRSA cannot be defined only by previously specified genes. It also suggests that the risk of zoonotic transmission between production animals and humans together with the public health threat associated with MRSA in livestock, may be larger than previously assessed.

Together with previously published studies and surveillance data, the results in this study also confirms that the Norwegian LA-MRSA strategy is effective in identifying and controlling the spread of MRSA to and between pig herds, and that we so far have managed to prevent MRSA in livestock from becoming a domestic source of MRSA transmission to the public. If only few and sporadic cases of MRSA in livestock continue to emerge, the current strategy and control guidelines against MRSA in the pig population should be maintained instead of implementing comprehensive and resource demanding prevention against domestic LA-MRSA in hospitals. Continuing success of the national MRSA strategy will prevent LA-MRSA related disease in the Norwegian public.

## Author Contributions

PE, CAG, ØA, SÅ, AU, and SJ performed the outbreak investigations; PE, CAG, SÅ, AU, and SJ collected the data; PE, CAG, CG, MSteg, HE, AU, MStei, and MSu analyzed and interpreted the data; PE, CAG, and CG prepared the tables and figures; PE and CAG contributed equally, with the primary responsibility of writing and revising the manuscript; MStei and MSu contributed equally; and all authors contributed to revising the manuscript and approved the final version.

### Conflict of Interest Statement

The authors declare that the research was conducted in the absence of any commercial or financial relationships that could be construed as a potential conflict of interest.
